# Porous Zinc Anode Design for Zn-air Chemistry

**DOI:** 10.3389/fchem.2019.00656

**Published:** 2019-10-01

**Authors:** Peiyuan Liu, Xiaofei Ling, Cheng Zhong, Yida Deng, Xiaopeng Han, Wenbin Hu

**Affiliations:** ^1^Tianjin Key Laboratory of Composite and Functional Materials, School of Materials Science and Engineering, Tianjin, China; ^2^Key Laboratory of Advanced Ceramics and Machining Technology, Ministry of Education, Tianjin University, Tianjin, China; ^3^Joint School of National University of Singapore and Tianjin University, International Campus of Tianjin University, Fuzhou, China

**Keywords:** Zn-air battery, Zn anode, porous Zn, electrochemical activity, anti-corrosion

## Abstract

Zinc-air battery has drawn increasing attention from the whole world owing to its large energy capacity, stable working voltage, environmentally friendship, and low price. A special porous Zn with three-dimensional (3D) network frame structure, whose multistage average pore sizes can be tuned from 300 to 8 um, is synthesized in this work. It is found that there is a competition between Zn^2+^ and ${\text{NH}}_{4}^{+}$ for their reduction on the supports. And the decrease of Zn^2+^ concentration and increase of ${\text{NH}}_{4}^{+}$ concentration can facilitate the decrease of pore size. Potential-dynamic polarization was tested with 3-electrodes cell, aiming to characterize the electrochemical activity and corrosion properties of porous Zn and commercial Zn foil electrodes. After optimization, the porous Zn prepared with the parameters of 3 M NaBr, 1 M C_2_H_3_O_2_NH_4_, and 0.01 M C_4_H_6_O_4_Zn shows the most negative corrosion potential of −1.45 V among all the samples, indicating the remarkable anti-corrosion property. Its discharge specific capacity is up to 812 mAh g^−1^. And discharge-charge test of the porous Zn shows an initial discharge platform of 1.33 V and an initial charge platform of 1.96 V, performing a small overpotential. What's more, the porous Zn exhibits a much longer cycle life than commercial Zn foil. Our work will not only shed light on the design and synthesis of other porous metal materials, but also further promote the development of Zn-based battery electrochemistry.

## Introduction

The past 20 years have witnessed the transforming of global energy industries. The fast consumption of fossil energies (coal, petroleum, and natural gas) and the accompanied environmental pollution have drown people's attention to ecological improvement and the utilization of green energy resources like solar energy, tidal energy, nuclear power, wind energy, etc (Fu et al., 2017; Mainar et al., 2018). However, these multiple powers have their own limitations on generations, transportations, or especially safety. Nowadays, the trend of energy storage and utilization has been dominated by secondary batteries, for instance, Ni-Zn cell, Ni-Cd cell, and Li-ion cell (Zhang et al., 2012, 2019; Moser et al., 2013; Assefi et al., 2018; Varzi et al., 2018; Hu et al., 2019). And Li-based battery has been promoted all over the world, on account of its stable voltage window, high working voltage, energy density of a high degree, low self-discharge, and high reversibility (Zhang et al., 2012, 2019; Varzi et al., 2018; Hu et al., 2019). Unfortunately, troubles in the general application such as high cost, short circuit, dendrites, and other safety problems (kindling and exploding) have restrict the development of Li-based battery (Lisbona and Snee, 2011; Shi et al., 2019). To solve these troubles, some security mechanisms for over-charge and over-discharge have been applied to Li-based cell systems, which would further increase the cost and let the energy storage and appliance facilities to be more burdensome (Lisbona and Snee, 2011). Therefore, it is inevitable and urgent to find some replacements to Li-based battery systems (Li and Dai, 2014; Fu et al., 2017; Wang et al., 2017).

Zn-air secondary battery has superiorities of large capacity, high specific energy (its theoretical mass ratio energy is 1,086 Wh kg^−1^), stable working voltage, environmentally friendship, no pollution, easy availability of raw materials, and low in price (Vatsalarani et al., 2005; Cho and Fey, 2008; Lee et al., 2013; Li and Dai, 2014; Fu et al., 2017; Wang et al., 2017; Lu et al., 2018; Schmid and Willert-Porada, 2018). In recent years, it has attracted the growing concerns from research institutions and enterprises all over the world. As Zn-air battery is known as “the twenty first century's green new energy,” it is our expectation that zinc-air battery can accord with the development tendency of the new energy industry and meet the needs of the society. Its application range will become increasingly extensive, and it is expected to replace or partially displace Li-based battery systems (Li and Dai, 2014; Gu et al., 2017; Wang et al., 2017).

To achieve the conversion between electrical energy and chemical energy, metal zinc electrode dissolves into the electrolyte or Zn^2+^ deposits on the Zn electrode, and at the same time, the oxygen reduction reaction (ORR) or oxygen evolution reaction (OER) happens on the oxygen diffusion electrode (ODE). As presented in Equations (1) and (2), during the discharge of alkaline Zn-air cell, Zn dissolves into electrolyte, and O_2_ get reduced on ODE.

Negative (anode) electrode:

(1) $$\begin{array}{l} \text{Zn}+4\text{O}{\text{H}}^{-}-2{\text{e}}^{-}\to \text{Zn}{\left(\text{OH}\right)}_{4}^{2-}\\ \text{Zn}{\left(\text{OH}\right)}_{4}^{2-}\to \text{ZnO}+{\text{H}}_{2}\text{O}+{2\text{OH}}^{-}\;{\text{E}}^{\circ }=-1.25\text{ V vs}.\text{ SHE} \end{array}$$

Positive (cathode) electrode:

(2) $${\text{O}}_{2}+2_{\text{H2}}\text{O}+{\text{4e}}^{-}\to {\text{4OH}}^{-}\;{\text{E}}^{{}^{{}^{\circ}}}=0.4\text{ V vs}.\text{ SHE}$$

Overall reaction:

(3) $$\begin{array}{l} 2\text{Zn}+{\text{O}}_{2}\to 2\text{ZnO }{\text{E}}^{\circ }=1.65\text{ V} \end{array}$$

Hydrogen evolution self-corrosion of Zn anode (parasitic reaction):

(4) $$\begin{array}{l} \text{Zn}+2{\text{H}}_{2}\text{O}\to \text{Zn}{\left(\text{OH}\right)}_{2}+{\text{H}}_{2} \end{array}$$

In the last few years, there has been a lot of works focusing on positive electrode, and that helps to bring about the flourishing of OER and ORR catalyst (Han et al., 2014, 2018; He et al., 2017; Wu et al., 2017; Zhao et al., 2019). What's more, there are also many fundamental problems on the Zn anode side that must be addressed. From Equation (4), we can see that there is a parasitic reaction, the self-corrosion of Zn anode, which inevitably decrease the utilization of activated metallic zinc. Besides, Zn(OH)_2_ resolves into ZnO and H_2_O. It's accepted that ZnO not only be unable to show well electronic conductivity, but also reduce the contact surface area between Zn electrode and electrolyte (Gu et al., 2017). The decreased utilization of Zn will result in the loss of discharge capacity. Moreover, during charge, the dendritic crystallization may generate on the surface of Zn electrode and puncture the diaphragm between negative electrode and positive electrode, leading to the short circuit problem. In recent years, many researchers have paid attention on the methods of how to inhibit the self-discharge introduced by self-corrosion (Zhang et al., 2001; Cho and Fey, 2008; Lee et al., 2013; Sun et al., 2017). And the means of restraining the generation of dendrites and protrusions were explored to inhibit the shape change and to enhance the cycling stability (Zhang et al., 2001; Vatsalarani et al., 2005; Sun et al., 2017). From their works, adding additives (like Al_2_O_3_ and Bi_2_O_3_) or surface coating were found to be effective strategies to enhance the performance of Zn electrode. And it was reported that zinc particles anode coated with bismuth oxide based glasses was well functioned as anode to strengthen the reversibility (Schmid and Willert-Porada, 2018). Apart from the above mentioned treatments, the Zn electrode with large specific surface area can perform improved activities. And the enlargement of specific surface area of Zn electrode makes it effective in the inhibition of the performance degradation caused by passivation.

Keeping in mind of the above considerations, one kind of porous Zn electrode with stupendous specific surface area is designed in this work. The developed synthetic route is the bubble template assisted electrodeposition method, which has been applied to prepare porous Cu-based or noble metal materials (Shin et al., 2003; Shin and Liu, 2004; Li et al., 2007; Cherevko and Chung, 2010; Cherevko et al., 2010; Varzi et al., 2018; Qiu et al., 2019). The bubble template method is explored here for a new 3D porous Zn, and the synthesized porous Zn is used in alkaline Zn-air battery for the first time. The chemical mechanism of bubble template method has been systematically discussed in this study. C_4_H_6_O_4_Zn, C_2_H_3_O_2_NH_4_, and NaBr are used to prepare porous zinc. ${\text{NH}}_{4}^{+}$ functions as the source of H according to former studies (Shin et al., 2003; Shin and Liu, 2004; Li et al., 2007; Cherevko and Chung, 2010; Aldama et al., 2019; Qiu et al., 2019), the function of C_2_H_3_${\text{O}}_{2}^{-}$ is to decrease the size of pores, and NaBr is introduced to strengthen the conductivity of solutions. Detailed physicochemical characterizations reveal that porous zinc with unique foam structure is successfully fabricated using the dynamic gas template assisted electrodepositon approach. This novel foam structure is full of inter-connected pores, making it convenient for the mass transport and contributing to the accelerated electrochemical reaction kinetics in Zn-air chemistry. Our work provides a convenient and efficient synthetic method for porous Zn, and the study will have a constructive guiding effect on future researches and promote the application of Zn-based batteries.

## Experimental Section

### Regents and Materials

Commercial Zn foil and commercial Cu foil of high purity (yan hui science and technology Ltd., thickness was 0.3 mm) were polished on the grinding and polishing machine and cleaned in ultrasonic cleaning machine with acetone and dilute hydrochloric acid for 20 min. And Cu was foam obtained from Kunshan GuangJiaYuan new materials Co., Ltd. The Zn foils, Cu foils, and foam Cu after treatments were cut into 1 × 2 cm^2^. The chemicals, obtained from Shanghai Aladdin Biochemical Technology Co., Ltd., were shown as the follows: zinc acetate (C_4_H_6_O_4_Zn, 99%), ammonium acetate (C_2_H_3_O_2_NH_4_, AR, 99%), sodium bromide (NaBr, AR, 99%), and potassium hydroxide (KOH, AR, 99%). These drugs were dissolved in DI water. Platinum on graphitized carbon (Pt/C, 20% w/w Pt) was bought from Shanghai Hesen Biotechnology Co., Ltd. Nafion perfluorinated resin solution and absolute ethanol (99.7%) were purchased from Sigma-Aldrich (St. Louis, MO, USA) and Tianjin Kemiou Chemical Reagent Co., Ltd., respectively. To obtain Pt/C catalyst, the composition of 10 mg Pt/C, 450 uL DI water, 450 uL absolute ethanol, and 100 uL Nafion were mixed. Then the mixture was put into ultrasonic cleaning machine for 20 min. After ultrasonic process, the mixture turned into Pt/C slurry, and Pt/C slurry was dropped on carbon cloth (2 × 2 cm^2^).

### Preparation of Porous Zn Electrodes

The concentration of NaBr was set at 3 M to be a fixed value, and the concentrations of C_4_H_6_O_4_Zn and C_2_H_3_O_2_NH_4_ were set as two variants. When the concentration of C_4_H_6_O_4_Zn was changed, the concentration of C_2_H_3_O_2_NH_4_ was set at 1 M. And when the concentration of C_2_H_3_O_2_NH_4_ was changed, the concentration of C_4_H_6_O_4_Zn was set at 0.01 M. 3-electorde cell was used for the electrodeposition of foam Zn, with commercial Zn foil (1 × 2 cm^2^) as work electrode, Pt plate (1 × 1 cm^2^) as counter electrode, and saturated calomel electrode (SCE) as reference electrode. The distance between anode and cathode was 3 cm. The constant current of −5 A cm^−2^ was applied to the support, and the duration was set at 1 min at room temperature. The Supplementary Video of electrodeposition has been shown in Supplementary Material. Then to totally remove the impurity ions, the deposited porous Zn was cleaned by immersing into DI water and absolute ethanol for 10 min, respectively. After cleaning, the porous Zn was dried out by cold winds. The particular parameters of each sample are shown in Table 1, and the samples are named as Zn0.2, Zn0.1, Zn0.05, Zn0.01, NH_4_2, NH_4_1, NH_4_0.5, and NH_4_0.1, respectively.

**Table 1:** Different electrodepositing solutions and the corresponding deposited weight of porous Zn.

**Sample**	**NaOH (mol L^−1^)**	**C_4_H_6_O_4_Zn (mol L^−1^)**	**C_2_H_3_O_2_NH_4_ (mol L^−1^)**	**Deposited weight of Zn (g)**
1	3	0.2	1	1.26
2	3	0.1	1	0.767
3	3	0.05	1	0.366
4	3	0.01	1	0.014
5	3	0.01	2	0.008
6	3	0.01	1	0.014
7	3	0.01	0.5	0.047
8	3	0.01	0.1	0.003

*Samples 1–4 are for the studies of Zn^2−^ concentration; Samples 5–8 are for the studies of ${\text{NH}}_{\text{4}}^{+}$ concentration*.

### Materials Characterization

The morphologies of porous Zn anodes were studied by scanning electron microscopy(SEM, S4800 Hitachi, 30 kV) and scanning electron microscopy (SEM, JSM-7800F JEOL, 30 kV), and the observation of the samples by scanning electron microscopy of Hitachi and JEOL were operated at 15 and 5 kV, respectively. The structural studies and component analysis were carried out with X-ray diffraction (XRD). The operation was implemented with Cu-Kα radiation (λ = 0.154 nm). And specimens were tested with the range of 2θ from 10° to 90° at the scanning rate of 5° min^−1^.

### Electrocatalytic Measurements

#### The Electrochemical Tests for Zn Electrodes

The electrochemical tests for Zn electrodes, aiming to characterize their anti-corrosion properties and electrochemical activities, were carried out using an electrochemical workstation (iviumstat electrochemical interface) at room temperature. The 3-electrodes cell was used for collecting potential-dynamic polarization and electrochemical impedance spectroscopy (EIS) curves. For these tests, the pure Zn or electrodeposited foam Zn of 2 cm^2^ functioned as work electrode, and the Pt plate (99.99%) of 1 cm^2^ was used as counter electrode. The distance between the work electrode and the counter electrode was about 3 cm. Hg/HgO electrode was chosen as reference electrode. And the solution was 6 M KOH. For potential-dynamic polarization, the potential range was set from −1.25 V to −1.5 V vs. Hg/HgO, and scan rate was 50 mV s^−1^. For EIS measurements, the potential was set at open circuit potential (OCP), and the high frequency and low frequency were 100 kHz and 0.01 Hz, respectively.

#### Performance Testing of Zn-air Batteries

A homemade Zn-air cell model was assembled with pure Zn or porous Zn (1 × 2 cm^2^) as anode, Pt/C catalyst as cathode, and 6 M KOH as electrolyte. The loading amount of Pt/C on carbon cloth was 1.5 mg cm^−2^. And the weight of porous Zn electrodeposited on the support was 0.14 g. The Zn-air tests were carried out by a battery tester (LANHE CT2001A) at room temperature. Both discharge and discharge-charge curves were tested at 5 mA cm^−2^. The current densities of discharge tests at different current densities were set at 1, 2.5, 5, and 10 mA cm^−2^ to measure the rate performance. Before all of the tests, the introduction of high-purity O_2_ (Air Product, purity 99.995%) were needed for 30 min to make sure the solutions were saturated of oxygen. The devices must be kept at open circuit potential (OCP) for 5 min to make sure the voltage between the two electrodes becomes stable before testing.

## Results and Discussion

**Figure 1:**
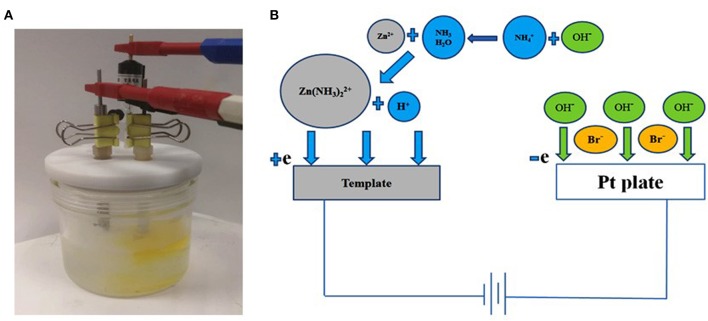
The images of reaction device and the reaction process. **(A)** reaction device, **(B)** reaction process.

Figure 1A shows the three-electrode configuration for the preparation of porous Zn. The electro-deposition processes for porous Zn are shown in Figure 1B. During the deposition of Zn foam, there were plenty of gases generating from both the work and counter electrode, and the solution around the counter electrode turned yellow, indicating the generation of Br_2_ (Supplementary Video 1). The experimental phenomena should be attributed to the reduction of $\text{Zn}{\left({\text{NH}}_{3}\right)}_{2}^{2+}$ and H^+^ around the cathode, and the oxidation of OH^−^ and Br^−^ close to the anode. Around the cathode, firstly, ${\text{NH}}_{4}^{+}$ hydrolyzed to NH_3_·H_2_O and H^+^. Next, $\text{Zn}{\left({\text{NH}}_{3}\right)}_{4}^{2+}$ formed via complexation between NH_3_·H_2_O and Zn^2+^ (Yang and Tang, 2000; Ruiz et al., 2007; Yang et al., 2019). But it was hard for $\text{Zn}{\left({\text{NH}}_{3}\right)}_{4}^{2+}$ to get electrons. Thus, $\text{Zn}{\left({\text{NH}}_{3}\right)}_{4}^{2+}$ lost 2 NH_3_ molecules and transformed into $\text{Zn}{\left({\text{NH}}_{3}\right)}_{2}^{2+}$, and H^+^ turned into H_2_ bubbles. Then, the porous Zn formed by electrodeposition process around these hydrogen bubbles.

**Figure 2:**
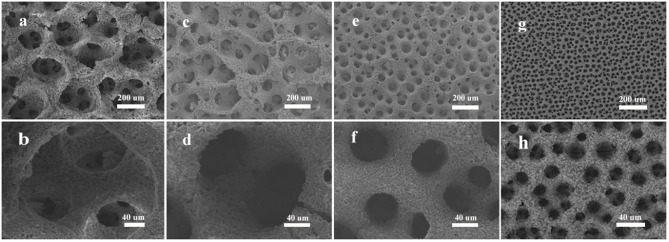
Electrodeposited 3D porous zinc structures at different concentrations of Zn^2+^: **(a,b)** 0.2 M, **(c,d)** 0.1 M, **(e,f)** 0.05 M, **(g,h)** 0.01 M. NaBr and C_2_H_3_O_2_NH_4_ were 3 M and 1 M, respectively.

As shown in SEM images (Figure 2), a series of porous Zn with different size of pores was prepared at different Zn^2+^ concentrations. The concentrations of C_4_H_6_O_4_Zn were selected at 0.2, 0.1, 0.05, and 0.01 M while the other conditions were kept at the same values (1 M C_2_H_3_O_2_NH_4_ and 3 M NaBr). It is easy to find that the pore size of the synthesized Zn electrode becomes smaller with the decreased concentration of C_4_H_6_O_4_Zn. From the enlarged magnitude images (Figures 2b,d,f,h), we can see that the holes for different concentrations are about 300, 130, 40, and 12 um on average for Zn0.2, Zn0.1, Zn0.05, and Zn0.01, respectively. Thus, the reduced concentrations of Zn^2+^ can let the pore sizes to be smaller. Figures 2c–f apparently show multi-scale porous structures with smaller holes generated on the walls of larger ones, which may lead to efficient mass transfer. The porous structures formed on the walls should be caused by the hydrogen bubbles that initiated on not only substrate but also the walls. The porous frameworks above show largely increased specific surface areas, potentially contributing to enhanced reaction rates. As shown in Table 1, we can find that the mass of final electrodeposited Zn will decrease with the decreased Zn^2+^ concentration at the same electrodeposition time of 2 min.

**Figure 3:**
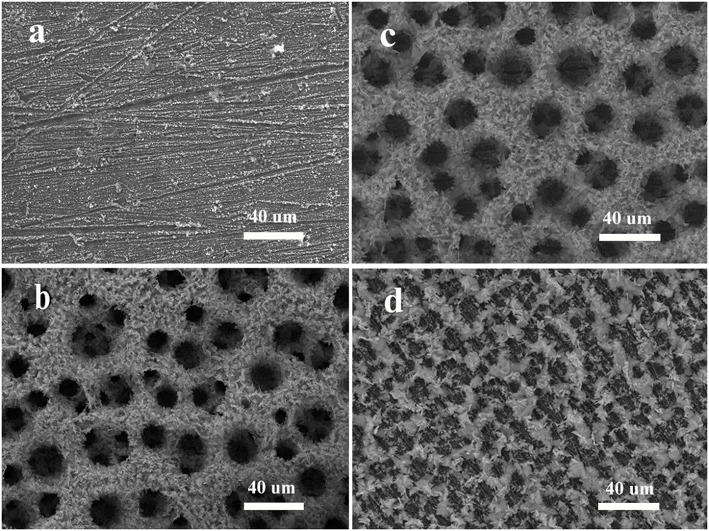
SEM images of electrodeposited Zn for the influence of ${\text{NH}}_{4}^{+}$: **(a)** 0.1 M, **(b)** 0.5 M, **(c)** 1 M, **(d)** 2 M. NaBr and C_4_H_6_O_4_Zn were 3 M and 0.01 M, respectively.

We then investigate the effect of ${\text{NH}}_{4}^{+}$ on the formation of the synthesized Zn materials. The results are displayed in Figure 3. When the concentration of ${\text{NH}}_{4}^{+}$ is 0.1 M, there is almost no product formed on the support. And when ${\text{NH}}_{4}^{+}$ concentration increases from 0.5 to 1 M, and eventually to 2 M, the number of pores also raises, and the average size of pores decreases from 20, to 12 um, and to 8 um. According to Figure 3a, when the concentration of ${\text{NH}}_{4}^{+}$ is too low, it is difficult for Zn^2+^ to deposit on the templates. As shown in Figure 3b, when the concentration is over some critical value, 0.5 M in our case, the pores are getting smaller with the increasing of ${\text{NH}}_{4}^{+}$ concentration. In Figure 3c, we can see that the pores of NH_4_1 are larger than that of NH_4_2 (Figure 3d). The weights of electrodeposited Zn at different concentrations of ${\text{NH}}_{4}^{+}$ are shown in Table 1. Obviously, the deposited mass of NH_4_0.1 is the least, owing to the insufficient of ${\text{NH}}_{4}^{+}$ and thus less $\text{Zn}{\left({\text{NH}}_{3}\right)}_{2}^{2+}$. Besides that, with the increase of ${\text{NH}}_{4}^{+}$ concentration, the weight of final electrodeposited Zn would decrease, which is attributed to the abundance of H^+^ and the lack of Zn^2+^ (this will be discussed latter). So we can make sure that in our system, the existence of ${\text{NH}}_{4}^{+}$ plays a vital role in the growth and the structure control of electrodeposited Zn metal.

**Figure 4:**
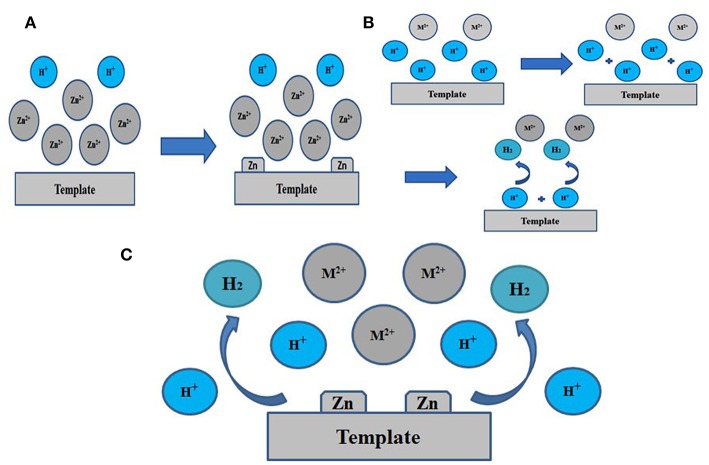
Schematic diagram for the preparation procedure. **(A)** for excessive Zn^2+^, **(B)** for excessive ${\text{NH}}_{4}^{+}$, **(C)** for appropriate ratio. M^2+^ is $\text{Zn}{\left({\text{NH}}_{3}\right)}_{2}^{2+}$.

More objective and comprehensive explanations have been provided to explain the above results. Our studies reveal that both Zn^2+^ and ${\text{NH}}_{4}^{+}$ ions have significant effects on the electrodeposition of porous Zn on substrate. As schematically illustrated in Figure 4A, when using a larger concentration of Zn^2+^, Zn^2+^ is so excessive that H^+^ is lack of chance to approach the electrode, leading to the abundance of Zn^2+^ and the insufficiency of H^+^ on the surface of support. There are only a small number of H_2_ bubbles that act as dynamic templates for the formation of Zn. Actually, if H^+^ is insufficient, H_2_ bubbles will be lack of driving force to get away from the support. Subsequently, these bubbles will gather together and turn into much larger size until adequate driving force are provided for desorption by the buoyancy of solution. In this case, Zn growing alongside these bubbles will form pores of much lager size, as shown in Figure 2a. On the contrary, we can further explain the effect of ${\text{NH}}_{4}^{+}$ (Figure 4B). It is found that zinc can hardly deposited in the case of NH_4_0.1 sample, revealing that $\text{Zn}{\left({\text{NH}}_{3}\right)}_{2}^{2+}$ is essential during the electrodeposited process. If ${\text{NH}}_{4}^{+}$ is sufficient, hydrogen bubbles will get enough driving force for desorption and won't aggregate, thus smaller pores will generate, consistent with experimental results in Figure 3. Figure 4C shows the case of appropriate proportion between Zn^2+^ and H^+^. Accordingly, a large number of Zn^2+^ will transfer into $\text{Zn}{\left({\text{NH}}_{3}\right)}_{2}^{2+}$. And it is clear that not only $\text{Zn}{\left({\text{NH}}_{3}\right)}_{2}^{2+}$ but also H^+^ will gets reduction on the support, turning into porous zinc and H_2_, respectively. In Figures 2c–f, 3b,c, we can see lots of small pores on the walls of larger pores, similar to the former research (Shin et al., 2003), which is due to the superior electronic conductivity of porous zinc metal. As shown in Figures 2g,h, 3d, the smaller holes on walls will disappear when the size of pores further decrease. Under these conditions, these pores further decrease to smaller than 10 um, which is not big enough for hydrogen bubbles' formation on the walls of pores. Generally speaking, $\text{Zn}{\left({\text{NH}}_{3}\right)}_{2}^{2+}$ has priority to Zn^2+^ for electrochemical reduction when ${\text{NH}}_{4}^{+}$ is sufficient (at least 1 M in our experiment). Moreover, the Zn electrodeposition competes with the hydrogen evolution, dramatically affecting the porous structure and the deposited mass of final Zn products (Table 1). We optimize that 3 M NaBr, 1 M C_2_H_3_O_2_NH_4_, and 0.01 M C_4_H_6_O_4_Zn is the best parameter with combined consideration of appropriate pore size and quantity of electrodeposited Zn. The effects of surfactants on the construction of porous Zn were also investigated by adding hexadecyl trimethyl ammonium bromide (CTAB). It is found that the addition of CTAB will results in the smaller pores of final Zn foams with dendrite-like structures formed on the surface (Figure S1), just like the former studies of porous Cu, Ag, and Au (Nikolic et al., 2007; Cherevko and Chung, 2010, 2011). Furthermore, to testify the universality of this method, the porous Zn was also electrodeposited on commercial Cu foil and foam Cu with the same parameter as Zn0.01. The SEM images of corresponding electrodeposited samples are shown in Figure S2, confirming the porous nanostructure of Zn electrodes.

**Figure 5:**
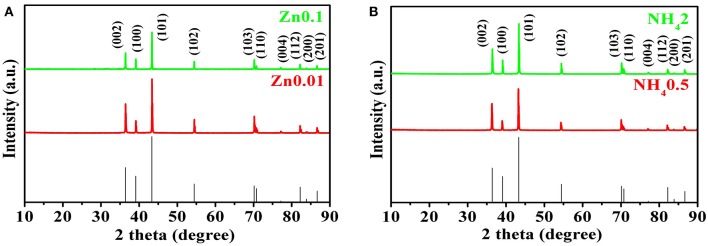
The XRD patterns for porous Zn of different concentrations. **(A)** for Zn0.1 and Zn0.01, **(B)** for porous Zn for NH_4_2 and NH_4_0.5.

**Figure 6:**
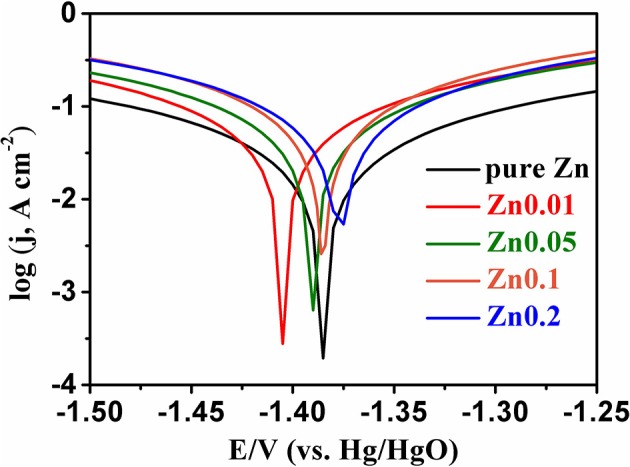
The dynamic-potential polarization curves for pure Zn and porous Zn prepared at different concentrations of Zn^2+^ in 6 M KOH with a scan rate of 50 mV s^−1^.

The XRD patterns are shown in Figure 5. All the peaks of selected Zn products are consistent with those of pure Zn (PDF card no. 65-5973) without any other peaks, indicating the high-purity of electroplated Zn. After electroplating of porous zinc, the samples should be cleaned and stored in absolute ethanol to prevent any possible oxidization. The hydrogen evolution self-corrosion of prepared Zn anode (shown in Equation 4) has been measured. Potential-dynamic polarization test was carried out to characterize the corrosion performance of commercial Zn foil and porous Zn with different pore sizes (Figure 6). The corrosion potential can be obtained by extrapolating the linear portion of the curves. Accordingly, the potential is −1.39 V for pure Zn while those of Zn0.01, Zn0.05, Zn0.1, and Zn0.2 are −1.45, −1.398, −1.388, and −1.375 V, respectively, suggesting that the synthesized Zn0.01 and Zn0.05 electrodes exhibit better anti-corrosion performance than that of pure Zn. Among these samples, Zn0.01 sample shows the most negative corrosion potential of −1.45 V, indicating the largest overpotential for HER. This means that the anti-corrosion properties can be influenced by the pore structure. Obviously, the pores formed with 0.2 M Zn^2+^ are averagely up to 300 um, which leads to the fast mass transportation and diffusion rate, contributing to the severe corrosion property. When the pore sizes diminished, specific surface area is increased alongside with the maintenance of high electrochemical activity. When the holes are narrowed from 300 to 130 um, to 40 um, and to 12 um on average, the corrosion products, such as $\text{Zn}{\left(\text{OH}\right)}_{4}^{2-}$, are possibly limited into these much smaller pores, resulting in better anti-corrosion performance. Our results herein reveal that the most superior anti-corrosion property is achieved by Zn0.01 sample. The electrochemical impedance spectroscopy (EIS) was carried out to compare the electrochemical activity between Zn0.01 and commercial Zn. According to Figure S3, the impedance of Zn0.01 (1.8 Ω) is much smaller than that of commercial Zn foil, suggesting the better electrochemical activity and faster reaction kinetics. Therefore, Zn0.01 was selected for subsequent Zn-air battery tests.

**Figure 7:**
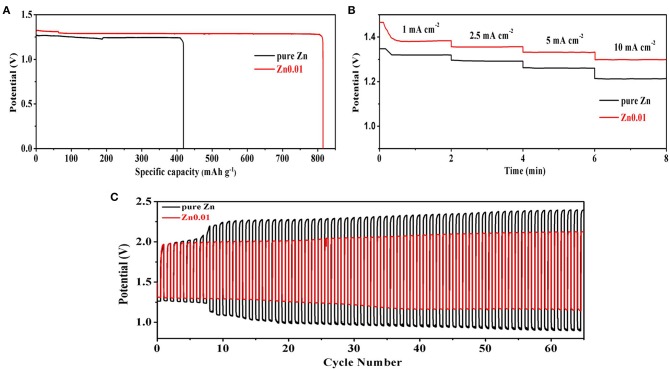
Zn-air tests of Zn0.01 and pure Zn foil: **(A)** discharge tests at 5 mA cm^−2^, **(B)** discharge tests at different current densities of 1, 2.5, 5, and 10 mA cm^−2^, **(C)** discharge-charge tests at 5 mA cm^−2^. The tests were carried out at room temperature.

Figure 7 illustrates the Zn-air performance of Zn0.01 and pure Zn. Figure 7A presents the discharge performance of commercial Zn and porous Zn at a current density of 5 mA cm^−2^. The starting voltage is 1.33 V for Zn foam while that is 1.26 V for pure zinc, indicating the high reactivity of the synthesized porous Zn electrode. Moreover, the specific discharge capacity of porous Zn is 812 mAh g^−1^, which is much larger than that of pure Zn (418 mAh g^−1^), suggesting the higher utilization ratio and energy efficiency of the former. This should be attributed to the fast mass transfer and electrochemical reaction kinetic brought by large specific surface area and large numbers of active sites. Figure 7B displays the rate discharge performance at different current densities (1, 2.5, 5, and 10 mA cm^−2^). Apparently, Zn-air battery based on Zn0.01 electrode shows higher discharge voltages than the cell using pure Zn at all current densities. Specifically, the discharge platforms of the former are 1.38, 1.36, 1.33, and 1.30 V at 1, 2.5, 5, and 10 mA cm^−2^, respectively, obviously outperforming those of the latter (1.32, 1.28, 1.26, and 1.22 V). This phenomenon again confirms the advantages of porous Zn in providing more electrochemical active sites and reducing reaction kinetics compared with Zn foil. The discharge-charge performance for rechargeable Zn-air batteries was further evaluated at 5 mA cm^−2^ (Figure 7C). The discharge voltage of Zn0.01 is 1.33 V and that is 1.26 V for pure Zn at the first cycle, and the charge voltage is 1.96 V for Zn0.01 and 2.28 V for pure Zn. Compared to Zn foil (1.02 V), the much smaller charge-discharge gap of Zn0.01 (0.63 V) indicates the high performance of Zn0.01 anode. Moreover, the discharge voltage of pure Zn decreases to 1.2 V at the 8th cycle while that of Zn0.01 delays to 33th cycle. As for the recharging voltage, both zinc foil and Zn0.01 start at 1.96 V, however, the Zn foil reaches 2.26 V after 8th cycles and 2.4 V after 65th cycles while that of Zn0.01 only increases to 2.1 V at the end. These results highlight the better performance of synthesized porous Zn foam electrode. In addition, as shown in Figure S4, the morphologies of pure Zn and Zn0.01 after discharge-charge test were characterized, revealing that Zn0.01 can partly maintain the porous structure after the discharge-charge test. It is obvious that Zn0.01 shows superior reversible performance than pure Zn. The superiority of that Zn electrode with abundant pores and high porosity is embodied in its high electrochemical activity, excellent reversibility, and better anti-corrosion.

## Conclusion

In this work, a kind of porous Zn with high activity is easily synthesized by bubble template method. We find that the performances of Zn-air battery can be raised by controllable pore coating on Zn electrode. The decrease of Zn^2+^ concentration and increase of ${\text{NH}}_{4}^{+}$ concentration can accelerate the decrease of pore size. More importantly, the decrease of pore size can strengthen the performance of Zn-air battery. When compared with commercial Zn foil, Zn0.01, prepared with the parameter of 0.01 M C_4_H_6_O_4_Zn, 1 M CH_3_COONH_4_, and 3 M NaBr, displays much higher specific capacity and discharge platform alongside with strengthened rate performance, increased cycle life and reduced overpotential. Therefore, our work has offered a novel method for the preparation of foam Zn for Zn-based battery chemistry.

## Data Availability Statement

The datasets generated for this study are available on request to the corresponding author.

## Author Contributions

PL conducted the experiments and write the manuscript. XL helped with operating the experiments and data analysis. CZ, YD, and XH interpreted the results. YD, XH, and WH supervised the research. All authors approved the submission of final manuscript.

### Conflict of Interest

The authors declare that the research was conducted in the absence of any commercial or financial relationships that could be construed as a potential conflict of interest.

## Supplementary Material

Supplementary Data Sheet 1 The SEM images of elctrodeposited porous Zn with the presence of CTAB, the SEM of porous Zn deposited on Cu foil and foam Cu, the EIS test of pure Zn and Zn0.01, and the SEM of pure Zn and Zn0.01 after discharge-charge tests.

Supplementary Video 1 The electrodeposition process of porous Zn.
