# Supplementary material for: Porous Zinc Anode Design for Zn-air Chemistry
Source: Front Chem. 2019 Oct 1;7:656. doi: 10.3389/fchem.2019.00656 (PMC6779696; doi:10.3389/fchem.2019.00656)
Supplement: Supplementary Data Sheet 1 — The SEM images of elctrodeposited porous Zn with the presence of CTAB, the SEM of porous Zn deposited on Cu foil and foam Cu, the EIS test of pure Zn and Zn0.01, and the SEM of pure Zn and Zn0.01 after discharge-charge tests. [file Data_Sheet_1.docx]

**Supporting Information**

Porous Zinc Anode Design for Zn-air Chemistry

Peiyuan Liu,*^1^* Xiaofei Ling,*^1^* Cheng Zhong,*^1,2^* Yida Deng,*^1,2^* Xiaopeng Han,^*^*^1,2^* Wenbin Hu*^1,2,3^*

^1^School of Materials Science and Engineering, Tianjin Key Laboratory of Composite and Functional Materials, ^2^Key Laboratory of Advanced Ceramics and Machining Technology (Ministry of Education), Tianjin University, Tianjin 300072, China; ^3^Joint School of National University of Singapore and Tianjin University, International Campus of Tianjin University, Binhai New City, Fuzhou 350207, China.


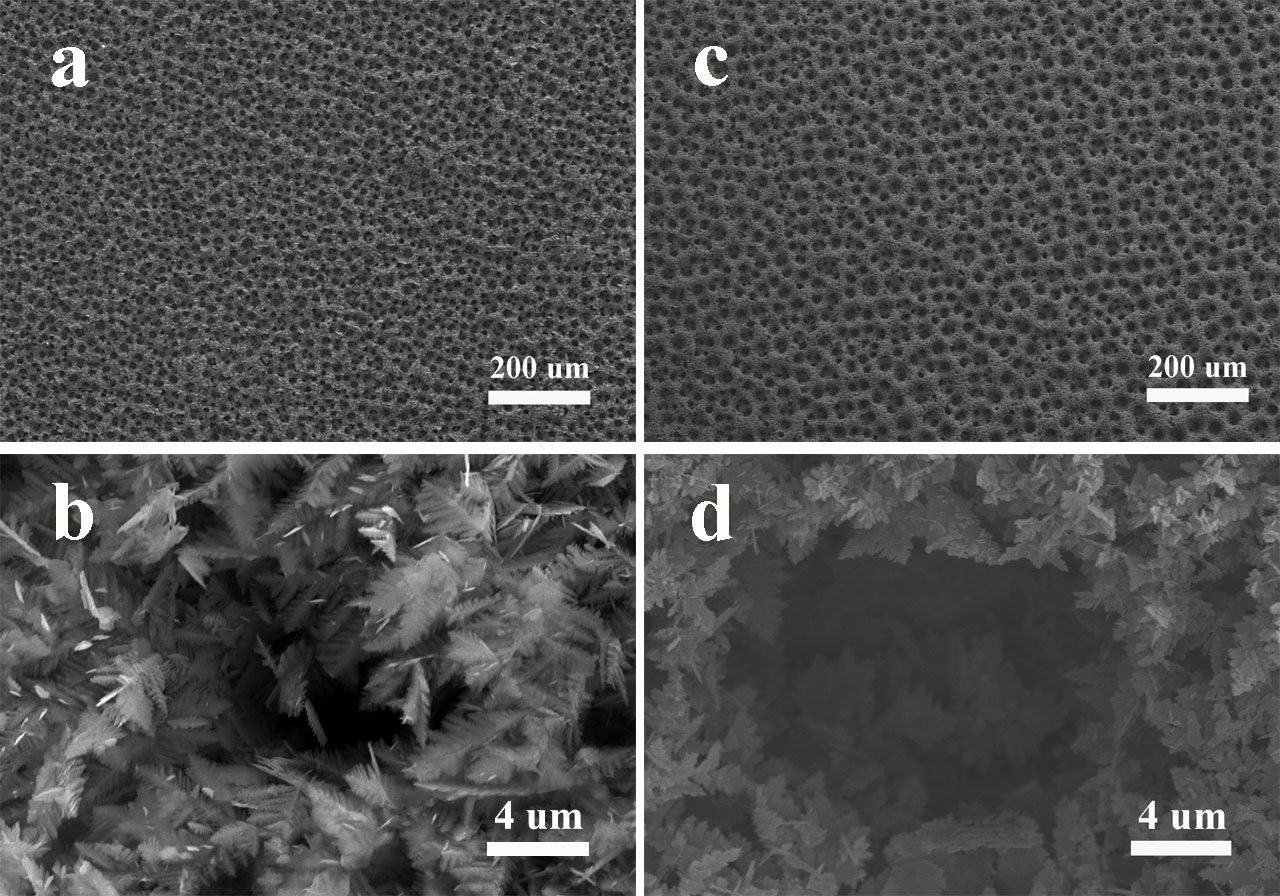


**Figure S1**. 3D porous zinc electrodepositing on the metal substrate for different concentrations of CTAB. (a, b) 0.1mM, (c, d) 0.05mM.The concentration of NaBr, C_4_H_6_O_4_Zn and CH3COONH_4_ were 3 M, 0.01 M and 1 M, respectively.

The effects of surfactants on construction of porous copper film by hydrogen bubble template method were studied ([Wang et al., 2011](#_ENREF_1)). To explore the influence of surfactant on porous Zn, hexadecyl trimethyl ammonium bromide (CTAB) was chosen. And the concentrations of CTAB were set at 0.1 mM and 0.05 mM. As shown in **Figure S1**, the pore size of porous Zn prepared at 0.1 mM CTAB (**Figure S1a, b**) is approximately 4 um, and that is about 8 um of foam Zn prepared at 0.05 mM CTAB (**Figure S1c, d**). Both pore sizes prepared at 0.1 mM and 0.05 mM CTAB are smaller than 12 um pores of Zn0.01 on average. Therefore, it’s apparently that the addition of CTAB is in favor of pore refinement of foam Zn. Further studies are needed to reveal the deeper mechanism.


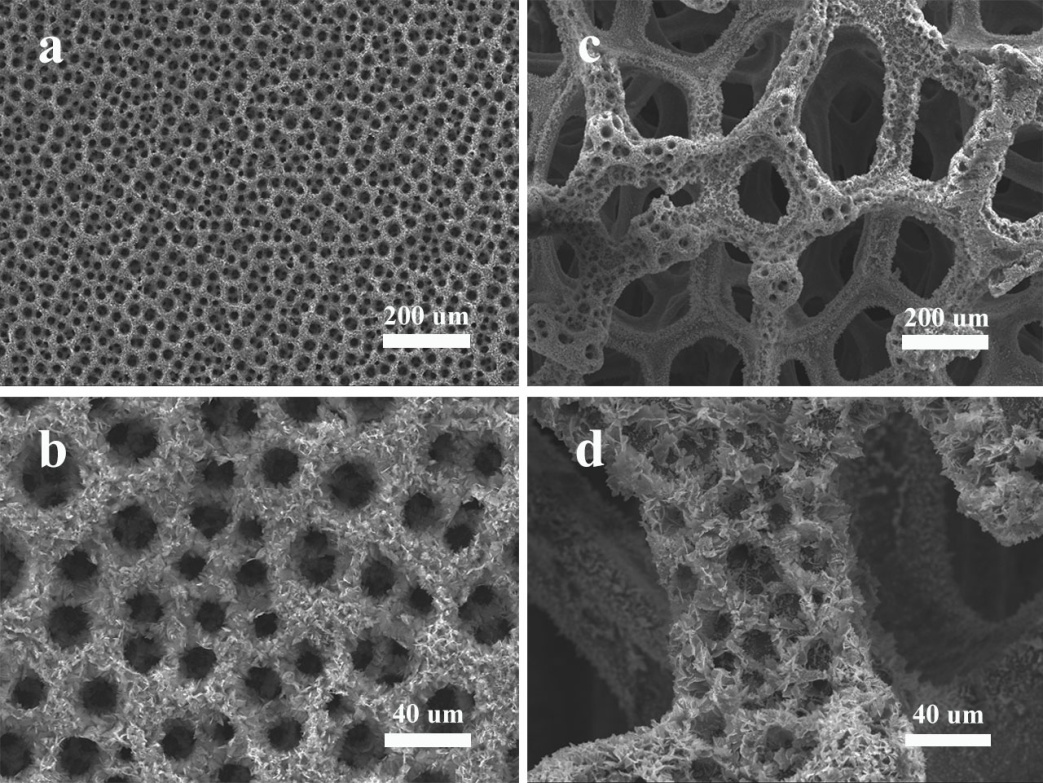


**Figure S2**. The SEM images of porous Zn deposited on other supports: (a, b) on Cu foil, (c, d) on Cu foam. The synthetic parameters were 3 M NaBr, 1 M C_2_H_3_O_2_NH_4_, and 0.01 M C_4_H_6_O_4_Zn.

As shown in **Figure S2**, the porous Zn also can be electrodeposited on other supports using our method, like Cu foil and Cu foam. According to **Figure S2a, b**, the porous Zn deposited on Cu foil shows the similar structure of Zn foam loaded on Zn foil. And in **Figure S2c, d**, we can see that the porous Zn is also loaded on Cu foam. But the porous structure disappears in the inner layers of foam Cu, which should be attributed to the insufficient electrodeposition time of 2 min.





**Figure S3**. The EIS tests of pure Zn and Zn0.01.

From electrochemical impedance spectra (Figure S3), we can see that porous Zn has a low impedance of 1.8 Ω, which is much smaller than that of commercial Zn foil, indicating the fast reaction kinetic property of the porous nanostructure.


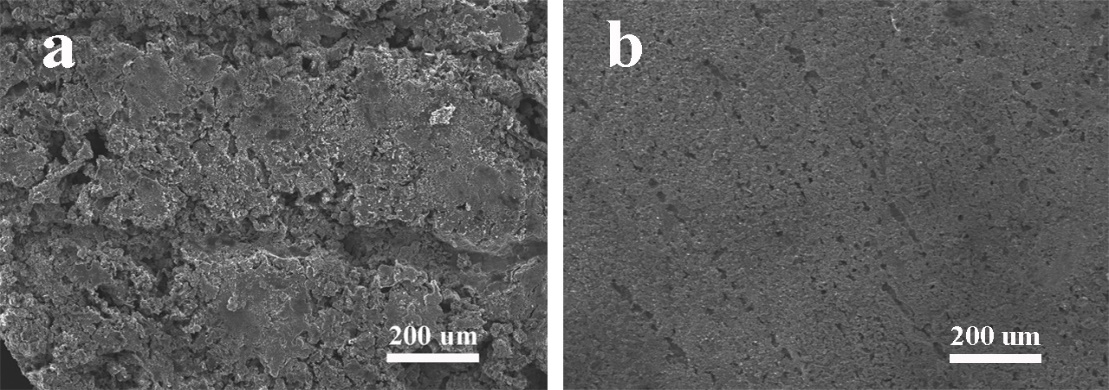


**Figure S4**. The Zn electrodes after discharge-charge tests. (a) pure Zn, (b)Zn0.01.

As shown in **Figure S4a**, the Zn foil after discharge-charge test has widened channels. These channels form in polishing process, and get corroded owing to the chemical and electrochemical reactions during test. **Figure S4a** display an apparent corrosion appearance. In **Figure S4b**, the channels of supporer are maintained, and porous structure remains on the carrier. The apparent comparasion between Zn foil and Zn0.01 further testify that Zn0.01 behaves obviously better in anti-corrosion.

**REFERENCES**

Wang, N., Hu, W. C., Lu, Y. H., Deng, Y. F., Wan, X. B., Zhang, Y. W., et al. (2011). Role of surfactants in construction of porous copper film by electrodeposition approach. *T I Met Finish.* 89(5), 261-267. doi: 10.1179/174591911x13119320025636.
